# Effects of Dietary Carbohydrate Levels on Growth Performance, Body Composition, Antioxidant Capacity, Immunity, and Liver Morphology in *Oncorhynchus mykiss* under Cage Culture with Flowing Freshwater

**DOI:** 10.1155/2022/7820017

**Published:** 2022-09-13

**Authors:** Wei Zhao, Han-Lin Wei, Zi-Qiao Wang, Xuan-Shu He, Jin Niu

**Affiliations:** State Key Laboratory of Biocontrol, Guangdong Provincial Key Laboratory for Aquatic Economic Animals and Southern Marine Science and Engineering Guangdong Laboratory (Zhuhai), School of Life Sciences, Sun Yat-Sen University, Guangzhou 510275, Guangdong Province, China

## Abstract

The purpose of this study is to investigate the effects of dietary carbohydrate levels on growth performance, body composition, antioxidant capacity, immunity, and liver morphology in *Oncorhynchus mykiss* under cage culture with flowing freshwater. Fish (initial body weight 25.70 ± 0.24 g) were fed five isonitrogenous (420 g/kg protein) and isolipidic (150 g/kg lipid) diets containing 50.6, 102.1, 151.3, 200.9 and 251.8 g/kg carbohydrate levels, respectively. The results indicated that fish fed diets containing 50.6-200.9 g/kg carbohydrate showed significantly higher growth performance, feed utilization, and feed intake than those fed 251.8 g/kg dietary carbohydrate levels. Based on the analysis of the quadratic regression equation for weight gain rate, the appropriate dietary carbohydrate requirement of *O. mykiss* was estimated to be 126.2 g/kg. 251.8 g/kg carbohydrate level activated Nrf2-ARE signaling pathway, suppressed superoxide dismutase activity and total antioxidant capacity, and increased MDA content in the liver. Besides, fish fed a diet containing 251.8 g/kg carbohydrate showed a certain degree of hepatic sinus congestion and dilatation in the liver. Dietary 251.8 g/kg carbohydrate upregulated the mRNA transcription level of proinflammatory cytokines and downregulated the mRNA transcription level of lysozyme and complement 3. Whole-body compositions were not affected by dietary carbohydrate levels. In conclusion, 251.8 g/kg carbohydrate level suppressed the growth performance, antioxidant capacity and innate immunity, resulting in liver injury and inflammatory response of *O. mykiss*. A diet containing more than 200.9 g/kg carbohydrate is not efficiently utilized by *O. mykiss* under cage culture with flowing freshwater.

## 1. Introduction

Feeds account for approximately 50-70% of production costs in intensive aquaculture, and dietary composition has a significant impact on the economic efficiency of fish production [1, 2]. Protein is considered to be the primary nutrient affecting the growth and health of fish, and it is also the most costly macronutrient in fish feed. Fishmeal is the preferred protein source for commercial aquafeeds because of its high protein content, good palatability, well-balanced amino acid composition, and high digestibility [3]. However, fish meal prices have risen by nearly 300% over the past decade due to increased demand and stagnant capture fisheries [4, 5]. Therefore, reducing the protein content in feed is essential for the sustainable development and higher profitability of the aquaculture industry. Increasing the proportion of nonprotein energy sources in aquatic feed, such as lipids and carbohydrates, is an important strategy to save protein, reduce the emission of nitrogen wastes, and reduce feed costs [1]. Carbohydrate is the most economical energy source and is widely used in aquatic feed to decrease feed costs and reduce protein catabolism as energy. However, fish, especially carnivorous, have low carbohydrate utilization [6, 7]. Currently, most studies have indicated that appropriate levels of dietary carbohydrates improved growth, antioxidant capacity, and resistance to pathogens of fish, and reduced the catabolism of proteins and lipids [8, 9]. On the contrary, excessive dietary carbohydrate intake caused metabolic disorder, poor growth performance, low nutrient utilization and suppress the immune response [10–13]. The carbohydrate requirements of fish are species-specific. Therefore, it is necessary to determine the maximum level of carbohydrates that can be used to promote growth and improve feed utilization in different fish species.

Rainbow trout (*Oncorhynchus mykiss*), as a carnivorous cold-water fish, has become one of the most important commercial cultured fish in the world. The annual output of *O. mykiss* in world aquaculture was 848.1 thousand tonnes in 2018 [14]. In recent years, freshwater cultured *O. mykiss* has become hot in China due to its fast growth, delicious meat, high nutritional value, and increased market demand. The annual production of *O. mykiss* in China was 37.84 thousand tonnes in 2020, of which Qinghai Province accounts for nearly half of the total production [15]. In China, especially Qinghai Province, cage culture with flowing water plays a dominant role in the freshwater culture of *O. mykiss*. Previous studies have reported that the requirements of dietary protein and lipids for *O. mykiss* cultured in an indoor circulating freshwater system were about 400-450 g/kg and 150-200 g/kg, respectively [16]. Brauge et al. [17] demonstrated that *O. mykiss* can effectively use carbohydrates for growth in seawater when the digestible carbohydrates are up to 250 g/kg in the diet. However, Hilton and Atkinson [18] reported a diet containing more than 140 g/kg carbohydrate is not efficiently utilized by *O. mykiss* cultured in freshwater. In addition, previous studies have also reported the effects of dietary carbohydrate levels or carbohydrate-to-protein ratio on feed utilization, feed intake, and glucose metabolism of *O. mykiss* [19–21]. Previous results showed that the carbohydrate requirement of *O. mykiss* was related to fish size, culture system, environment, and feed composition. Therefore far, the optimal dietary carbohydrate level for *O. mykiss* under cage culture with flowing freshwater remains unknown. Evaluating the optimum carbohydrate level is particularly important for further increasing the culturing scale of *O. mykiss* in China and reducing feed costs.

Based on the practical diet formulation, the present study was conducted using a fixed protein level of 420 g/kg and lipid level of 150 g/kg and graded carbohydrate levels to determine the optimal dietary carbohydrate level for *O. mykiss* under cage culture with flowing water. The effects of graded dietary carbohydrate levels on the growth performance, body composition, antioxidant capacity, immunity, and liver morphology of *O. mykiss* were investigated. The results of this study provide a reference for the commercial feed of *O. mykiss* under cage culture with flowing freshwater.

## 2. Materials and Methods

All animal care and experimental procedures in the present study were approved by the Institutional Animal Care and Use Committee (IACUC), Sun Yat-Sen University.

### 2.1. Experimental Diets

Five isonitrogenous (420 g/kg protein) and isolipidic (150 g/kg lipid) experimental diets were formulated to contain 50.6 (T1), 102.1 (T2), 151.3 (T3), 200.9 (T4) and 251.8 (T5) g/kg carbohydrate levels (Table 1). Corn starch was used as the dietary carbohydrate source. All ingredients were ground and sifted through a 100-mesh sieve. Then, all required ingredients were weighed and thoroughly mixed according to the dietary formula. Subsequently, distilled water (300 g/kg dry matter) was added to the mixture and stirred thoroughly. The mixtures were extruded with a puffing apparatus (Institute of Chemical Engineering, South China University of Technology, Guangdong, China) to produce 3.5-mm-diameter puffed pellets. Finally, all diets were dried in an air-conditioned room until the moisture level was less than 100 g/kg, and then stored at -20°C until used.

### 2.2. Fish and Experimental Conditions

*O. mykiss* were provided by the commercial company (Kaiteweide Ecological Fishery co., LTD, Qinghai, China). The experimental cage was placed in the upper reaches of the Yellow River (101.0′27′′E, 36.8′22′′N). Before the feeding trial, fish were cultured in cages (5 m ×5 m ×2 m) for 14 days to acclimate to the experimental conditions. During this period, fish were hand-fed the commercial diet containing 410 g/kg crude protein and 240 g/kg crude lipid (Aller Aqua, Qingdao, China) to satiation twice daily. After that, 450 fish (initial body weight 25.70 ± 0.24 g) were randomly stocked into 15 cages (2.8 m ×2.7 m ×2 m), for a stocking density of 30 fish per cage. Each experimental diet was randomly applied to triplicate cages. Fish were hand-fed to apparent satiation twice daily (07 : 00 and 17 : 00) for 42 days. Satiation feeding is based on the standard that the fish does not come up the water surface to eat the diet within half an hour. Daily feed consumption and number and weight of dead fish were recorded. During the feeding trial, the water temperature was 12-16°C and the dissolved oxygen was not less than 6.5 mg/L.

### 2.3. Sample Collection

After the feeding trail, all fish in each cage were fasted for 24 h. Then, in batches, all fish in each cage were anesthetized with 20 mg/L of tricaine methanesulfonate (MS-222, Sigma, USA). All fish in each cage were counted and individually weighed to evaluate growth performance. The liver from ten fish per cage were rapidly removed and washed with normal saline, and then stored -80°C for analysis of antioxidant parameters and gene expression [22]. Besides, from three fish randomly chosen from each cage, the liver was removed and fixed in 40 g/kg paraformaldehyde solution (Servicebio Technology Co., Ltd., Wuhan, China) for histological analysis. Finally, three fish per cage were randomly collected and stored -80°C for whole-body composition analysis.

### 2.4. Proximate Composition Analyses

Proximate composition of diets and whole-body composition of the fish were determined following the standard method described by AOAC [23]. The moisture was measured by drying to a constant weight in an oven at 105°C. The crude protein was determined using a Dumatherm nitrogen analyzer (Gerhardt GmbH & Co. KG，Germany) by Kjeldahl method after acid digestion, and protein was calculated as N ×6.25.

Crude lipid was measured using a Fat Soxhelt Extractor (Soxtec System HT6, Tecator, Höganäs, Sweden). The carbohydrate level in the diet was measured by the 3,5-dinitrosalicylic acid method [24].

### 2.5. Antioxidant Parameters Analysis

Liver samples were homogenized in ice-cold normal saline (1 : 10 dilution) and centrifuged at 3000 r/min (4°C) for 20 min to obtain the supernatant [25]. The superoxide dismutase (SOD) activity (Cat. No. A001-1), malondialdehyde (MDA) content (Cat. No. A003-1), and total antioxidant capacity (T-AOC) (Cat. No. A015-2) in the supernatant were measured using commercial kits (Nanjing Jiancheng Bioengineering Institute, Nanjing, China) based on the manufacturer's instructions.

### 2.6. Histological Observation

Liver samples were fixed in 40 g/kg paraformaldehyde solution (Servicebio Technology Co., Ltd., Wuhan, China) and then dehydrated in a graded ethanol series and embedded in paraffin. Sections (5 *μ*m thick) of the liver were obtained with a rotary microtome and stained with hematoxylin and eosin. Finally, the sections were observed and photographed using an optical microscope (Leica DMLB, Germany).

### 2.7. Real-Time Polymerase Chain Reaction (PCR) Analysis

Total RNA was extracted from the liver in each cage by a reagent kit (TaKaRa, Dalian, China). Agarose gel electrophoresis and spectrophotometer (NanoDrop 2000, Thermo Fisher, United States) were used to identify the quality and quantity of RNA. Then, the total RNA samples were diluted to the same concentration with diethylpyrocarbonate treated water for normalization. Subsequently, cDNA was synthesized by PrimeScript RT Reagent kit with gDNA Eraser (TaKaRa, Dalian, China) following the manufacturer's instructions. The real-time RT-PCR was carried out in a LightCycler 480 Real-Time System (Roche Applied Science, Basel Switzerland) with SYBR® Premix ExTaq™ II (TaKaRa, Dalian, China) according to the procedure described by Zhao et al. [26]. *β*-Actin was set as an endogenous control gene. The dissolution curve was used to detect the uniqueness of PCR products. The relative expression levels of target genes were calculated based on the 2^-*ΔΔ*CT^ method. The primer sequences for real-time RT-PCR were shown in Table 2.

### 2.8. Statistical Analysis

The specific growth ratio (SGR), survival rate (SR), weight gain rate (WGR), and feed conversion ratio (FCR) were calculated according to the equation described by Zhao et al. [26].

Feed intake (FI, %/day) =100 × dry matter weight of feed intake/[(final body weight + initial body weight)/2 × days].

The results were expressed as means ± standard error (SE) and analyzed using SPSS 20.0 statistical software (SPSS, Chicago, IL, USA). All data were checked for normality and homogeneity using the Kolmogorov-Smirnov test and Levene's test, respectively. The differences in data were analyzed by using one-way analysis of variance (ANOVA) followed by Tukey test. In addition, orthogonal polynomial contrasts were performed to test if the effect was linear and quadratic. *P* <0.05 was considered statistically significant.

## 3. Results

### 3.1. Biological Performance and Whole-Body Composition

**Figure 1:**
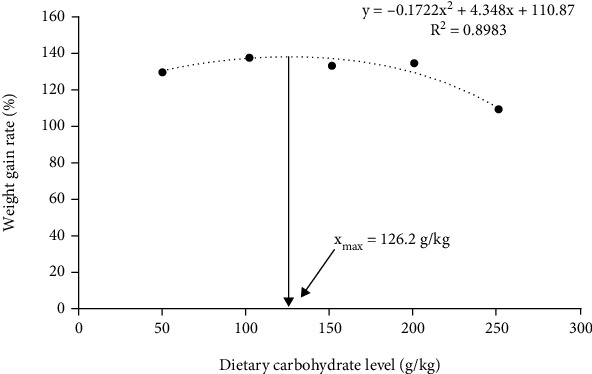
The dietary carbohydrate requirement based on weight gain rate (WGR) of *Oncorhynchus mykiss*.

The final body weight (FBW), SGR, WGR, FCR, and SR of *O. mykiss* fed with experimental diets were shown in Table 3. Results showed that significant quadratic (*P* <0.05) effects on FBW, SGR, WGR, and FI were observed in response to increased dietary carbohydrate levels. The FBW, SGR, WGR, and FI showed the lowest value in the T5 diet (*P* <0.05). There was no significant difference in the FBW, SGR, WGR, and FI among T1-T4 diets (*P* >0.05). The FCR was linearly (*P* <0.05) and quadratically (*P* <0.05) affected by dietary carbohydrate levels, and the highest value was observed in T5 diet (*P* <0.05). The SR was not affected by dietary carbohydrate levels (*P* >0.05) and ranged from 94.44 to 97.78%. Based on the analysis of the quadratic regression equation for WGR, the dietary carbohydrate requirement of *O. mykiss* under cage culture with flowing freshwater was 126.2 (Figure 1).

The crude protein, crude lipid, and moisture in the whole body were not affected by dietary carbohydrate levels (*P* >0.05) (Table 4).

### 3.2. Antioxidant Parameters

**Figure 2:**
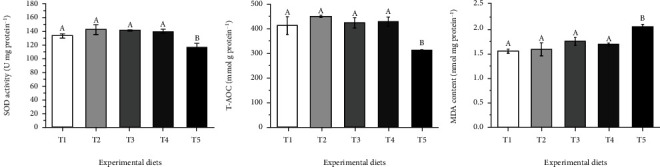
Effects of dietary carbohydrate levels on liver antioxidant parameters in *Oncorhynchus mykiss*. SOD: ANOVA, 0.02; Linear, 0.08; Quadratic, 0.00. T-AOC: ANOVA, 0.00; Linear, 0.02; Quadratic, 0.00. MDA: ANOVA, 0.00; Linear, 0.00; Quadratic, 0.00. Means with different superscripts are significantly different (*P* <0.05). Values are presented as mean ± SE, n =15.

As shown in Figure 2, the T-AOC and MDA content in the liver were linearly (*P* <0.05) and quadratically (*P* <0.05) affected by dietary carbohydrate levels. The lowest value of T-AOC and the highest value of MDA were obtained in T5 diet (*P* <0.05). The SOD activity in the liver was quadratically (*P* <0.05) affected by dietary carbohydrate levels, and the lowest value was observed in T5 diet (*P* <0.05). The T-AOC, MDA content, and SOD activity in the liver were not significantly changed among T1-T4 diets (*P* >0.05).

### 3.3. Morphological Observation of Liver

**Figure 3:**
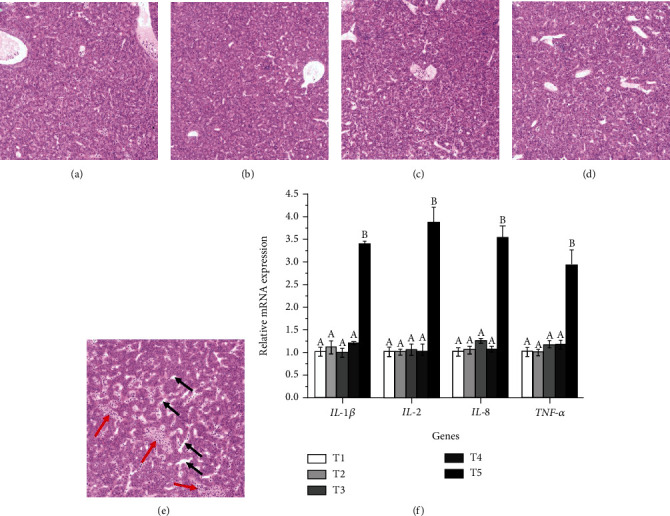
Liver morphology of *Oncorhynchus mykiss* fed with T1 (A), T2 (B), T3 (C), T4 (D) and T5 (E) diets, and relative expression levels of inflammation-related genes in the liver of fish fed experimental diets (F). The red arrow indicated hepatic sinus congestion. The black arrow indicated hepatic sinus dilatation. Means with different superscripts are significantly different (*P* <0.05). Values are presented as mean ± SE, n =15. *IL-1β*: ANOVA, 0.00; Linear, 0.00; Quadratic, 0.00. *IL-2*: ANOVA, 0.00; Linear, 0.00; Quadratic, 0.00. *IL-8*: ANOVA, 0.00; Linear, 0.00; Quadratic, 0.00. *TNF-α*: ANOVA, 0.00; Linear, 0.00; Quadratic, 0.00. Magnification 200 × .

As shown in Figure 3, T1-T4 diet groups showed a clear structure of the hepatic cord and intact and tightly arranged hepatic cells. The liver of fish fed with T1-T4 showed a healthy liver morphology. However, T5 diet group showed a certain degree of hepatic sinus congestion and dilatation in the liver.

### 3.4. Gene Expression Related to Inflammation, Antioxidant, and Immune in Liver

As shown in Figure 3(f), the mRNA levels of inflammation-related genes in the liver, including interleukin 2 (*IL-2*), interleukin 1*β* (*IL-1β*), interleukin 8 (*IL-8*) and tumor necrosis factor-*α* (*TNF-α*), were linearly (*P* <0.05) and quadratically (*P* <0.05) affected by dietary carbohydrate levels. Compared with T1-T4 diet group, the mRNA levels of *IL-2*, *IL-1β*, *IL-8,* and *TNF-α* were significantly upregulated in the T5 diet group (*P* <0.05).

**Figure 4:**
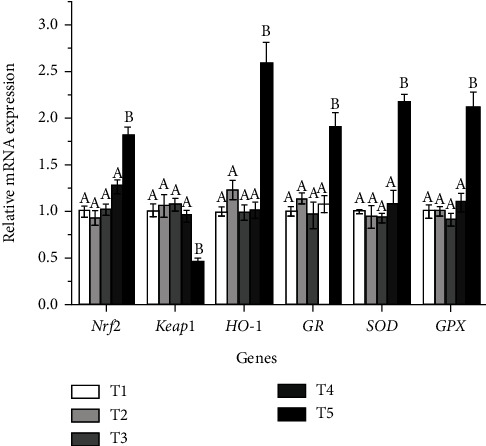
Relative expression levels of antioxidant-related genes in the liver of *Oncorhynchus mykiss* fed experimental diets. Means with different superscripts are significantly different (*P* <0.05). Values are presented as mean ± SE, n =15. *Nrf2*: ANOVA, 0.00; Linear, 0.00; Quadratic, 0.00. *Keap1*: ANOVA, 0.00; Linear, 0.01; Quadratic, 0.00. *HO-1*: ANOVA, 0.00; Linear, 0.01; Quadratic, 0.00. *GR*: ANOVA, 0.00; Linear, 0.01; Quadratic, 0.00. *SOD*: ANOVA, 0.00; Linear, 0.00; Quadratic, 0.00. *GPX*: ANOVA, 0.00; Linear, 0.00; Quadratic, 0.00.

The mRNA levels of antioxidant-related genes in the liver, including NF-E2-related nuclear factor 2 (*Nrf2*), Kelch-like ECh-associated protein 1 (*Keap1*), glutathione reductase (*GR*), haeme oxygenase-1 (*HO-1*), glutathione peroxidase (*GPX*) and *SOD*, were linearly (*P* <0.05) and quadratically (*P* <0.05) affected by dietary carbohydrate levels. Fish fed with T5 diet showed significantly higher mRNA levels of *Nrf2*, *GR*, *HO-1*, *GPX,* and *SOD* and lower mRNA levels of *Keap1* (*P* <0.05) (Figure 4).

**Figure 5:**
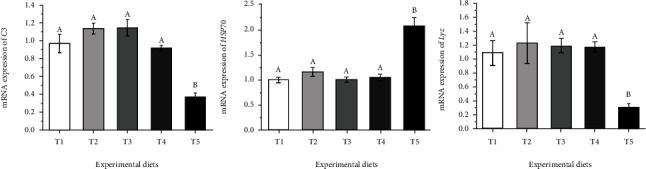
Relative expression levels of immune-related genes in the liver of *Oncorhynchus mykiss* fed experimental diets. Means with different superscripts are significantly different (*P* <0.05). Values are presented as mean ± SE, n =15. *C3*: ANOVA, 0.00; Linear, 0.01; Quadratic, 0.00. *HSP70*: ANOVA, 0.00; Linear, 0.01; Quadratic, 0.00. *Lyz*: ANOVA, 0.00; Linear, 0.01; Quadratic, 0.00.

The mRNA levels of immune-related genes in the liver, including complement 3 (*C3*), lysozyme (*Lyz*), and heat shock protein 70 (*HSP70*) were linearly (*P* <0.05) and quadratically (*P* <0.05) affected by dietary carbohydrate levels. Compared with T1-T4 diet group, the mRNA level of *HSP70* was significantly upregulated and the mRNA levels of *C3* and *Lyz* were significantly downregulated in the T5 diet group (*P* <0.05) (figure 5).

## 4. Discussion

Previous studies found that fish fed diets with excess carbohydrate levels exhibit significantly poorer growth performance than those fed with appropriate dietary carbohydrate levels, such as *Larimichthys crocea* [27], *Tilapia nilotica* [28], *Epinephelus akaara* [29] and *Rachycentron canadum* [30], which is mainly due to reduced FI and feed utilization [27, 30]. Similarly, the present study indicated that *O. mykiss* fed diets containing 50.6-200.9 g/kg carbohydrate showed significantly higher growth performance, feed utilization, and FI than those fed 251.8 g/kg dietary carbohydrate levels. Based on the analysis of the quadratic regression equation for WGR, the appropriate dietary carbohydrate requirement of *O. mykiss* was estimated to be 126.2 g/kg. Hilon and Atkinson [18] indicated that growth performance was significantly reduced in *O. mykiss* fed the diet containing 210 g/kg carbohydrate (*α*-glucose) in an indoor circulating freshwater system, and the excess 140 g/kg carbohydrate in the diet could not be effectively utilized by *O. mykiss*. Yilmaz [13] reported that 270 g/kg potato starch supplementation negatively affected growth performance (FCR, SGR and WGR) in *O. mykiss*. However, based on growth performance and feed efficiency ratios, a 400 g/kg protein diet with either 150 g/kg lipid and 180 g/kg gelatinized potato starch or 110 g/kg lipid and 270 g/kg gelatinized potato starch is appropriate for *O. mykiss* cultured in an indoor circulating freshwater system and self-feeding condition [31]. Brauge et al. [17] demonstrated that *O. mykiss* can effectively use carbohydrates (wheat starch) for growth in seawater when the digestible carbohydrates are up to 250 g/kg in the diet. Different carbohydrate sources affect the digestibility and efficient utilization of dietary starch by fish due to their different molecular structures, physical states, and content of inclusions [27]. Therefore, the current results indicate that the carbohydrate requirement of *O. mykiss* is related to fish size, carbohydrate source, culture conditions (salinity, temperature, culture system) and feeding method. In this study, a diet containing more than 200.9 g/kg carbohydrate is not efficiently utilized by *O. mykiss* under cage culture with flowing freshwater.

In the present study, whole-body compositions of *O. mykiss*, including crude protein, crude lipid, and moisture, were not affected by dietary carbohydrate levels. Similarly, Tekinay and Davies [21] found that the crude protein, crude lipid, and ash in the whole body and muscle of *O. mykiss* were not affected significantly by carbohydrate levels of the diet. Brauge et al. [17] suggested that the quantities of crude protein, crude lipid, moisture, and ash in the whole body of *O. mykiss* were not found to be different when the dietary digestible carbohydrate content was 80-244 g/kg. However, Ren et al. [30] demonstrated that *R. canadum* fed diets containing 125-304 g/kg cornstarch showed significantly higher crude lipid contents in whole body than those fed 13-65 g/kg cornstarch levels, which may be related to glycogen and lipid deposition in the liver caused by high carbohydrate intake. Li et al. [27] also observed that the crude lipid content of the whole body of *L. crocea* increased significantly with the increase of dietary carbohydrate levels. The different effects of dietary carbohydrate levels on the body composition of fish may be related to the degree of carbohydrate tolerance and metabolic mode of nonprotein energy sources of different fish species.

The observation of tissue morphology can intuitively show the health status of fish. In the present study, liver morphological examination showed pathological alterations in *O. mykiss* fed with a diet containing 251.8 g/kg carbohydrate level, including hepatic sinus congestion and dilatation. Similarly, excessive dietary carbohydrate levels resulted in hypertrophy of hepatocytes in *Labeo rohita* [32]. Similar observation has reported in *Megalobrama amblycephala* fed with a high carbohydrate diet [33]. Hepatocyte hypertrophy and sinus congestion are largely due to glycogen deposition in the liver induced by excessive carbohydrate [34]. Furthermore, the present study determined the effects of dietary carbohydrate levels on the mRNA levels of inflammation-related genes. In fish, inflammation is an important immune defense mechanism in response to tissue damage and infection [35]. Tissue damage and infection induce the activation and release of proinflammatory cytokines, such as *IL-1β*, *IL-2*, *IL-8,* and *TNF-α*, which mediate the onset of innate immune response [36, 37]. In this study, the mRNA expression levels of *IL-1β*, *IL-2*, *IL-8,* and *TNF-α* were significantly upregulated in fish fed a diet containing 251.8 g/kg carbohydrate level. This may be due to liver damage caused by dietary high carbohydrate levels. Excessive carbohydrate levels induce glycogen deposition in the liver, which leads to liver damage and further promotes the occurrence of an inflammatory response.

Nrf2-ARE pathway plays an important role in protecting cells from oxidative damage caused by endogenous and exogenous stress [38]. The antioxidant response element (ARE), an important transcriptional regulatory element, mediates the mRNA expression of a set of antioxidant factors, such as SOD, GPX, HO-1 and GR [22, 39]. Nrf2 is a key transcription factor that induces the expression of antioxidant factors regulated by ARE [40]. Under unstressed conditions, Keap1, a negative feedback regulator of Nrf2, binds to Nrf2 and accumulates in the cytoplasm as an inactive complex [41]. This quenching interaction inhibited the expression of Nrf2 and its regulated genes. However, under oxidative stress conditions, Nrf2 dissociates from Keap1 and subsequently binds to ARE in the nucleus to induce transcription of antioxidant factors [42]. In this study, *O. mykiss* fed a diet containing 251.8 g/kg carbohydrate levels upregulated the mRNA level of *Nrf2*, and downregulated the mRNA level of *Keap1* in the liver. Meanwhile, the mRNA transcription levels of antioxidant factors were significantly upregulated at 251.8 g/kg carbohydrate level, including *SOD*, *GPX*, *HO-1* and *GR*. The results indicated that dietary carbohydrate level of 251.8 g/kg resulted in oxidative stress in liver. Activation of the Nrf2/ARE pathway may be a response of hepatocytes to alleviate the damage caused by oxidative stress. Oxidative stress may increase free radical contents, resulting in increased lipid peroxidation [33]. Therefore, MDA content was measured to further confirm the degree of oxidative stress and lipid peroxidation in the liver. MDA, the product of lipid peroxidation, is an important indicator for evaluating the damage degree of cell structure and the degree of lipid peroxidation [43]. The results found that *O. mykiss* fed a diet containing 251.8 g/kg carbohydrate level showed significantly higher MDA content in the liver than those fed diets containing 50.6-200.9 carbohydrate levels. This also confirmed that a high-carbohydrate diet caused lipid peroxidation. Similar results were found in fish fed a high-carbohydrate diet, such as *M. amblycephala* [33], *Salvelinus fontinalis* [44] and *Micropterus salmoides* [11]. Besides, SOD activity and T-AOC in the liver were determined to evaluate to evaluate the effect of carbohydrate levels on the antioxidant capacity of *O. mykiss*. T-AOC is a critical index to reflect the total antioxidant capacity of fish [45]. The results found that SOD activity and T-AOC were significantly reduced in *O. mykiss* fed a diet containing 251.8 g/kg carbohydrate level. Similarly, Lin et al. [11] reported that *M. salmoides* fed a diet containing 200 g/kg starch level showed the lower SOD activity and higher MDA content in the liver than those fed diets containing 50-100 g/kg starch levels. It has also been reported that high-carbohydrate diet reduced SOD activity and increased MDA content in the liver of *Trachinotus ovatus* [9]. Liu et al. [46] found that *Erythroculter ilishaeformis* fed the diet containing 270 g/kg or 340 g/kg carbohydrate level had the lower SOD activity and T-AOC than those fed a diet containing 140 g/kg carbohydrate levels. Oxidative stress caused by high-carbohydrate diet may interfere with the metabolic system of fish, which led to reduction in activity of SOD [11]. In addition, excessive reactive oxygen species produced by oxidative stress led to liver injury, which may impair the release and activity of antioxidant enzymes. The present results suggested that high carbohydrate diet impaired the antioxidant capacity of *O. mykiss*, which may be attributed to the liver injury and antioxidant system imbalance caused by oxidative stress.

HSP70, a bioindicator for assessing stress status, can be activated by various environmental stressors, such as thermal shock, hypoxia, pollutants, and heavy metals [47]. HSP70 plays an important role in stress protection, increasing cell survival and improving tolerance to environmental stressors or injuries [48]. In this study, the mRNA transcription level of *HSP70* was significantly upregulated in *O. mykiss* fed a diet containing 251.8 g/kg carbohydrate level. Similar results have reported in *M. amblycephala* fed with a high carbohydrate diet [33]. The results indicated that high dietary carbohydrate led to oxidative stress, and the high expression of *HSP70* may be a response to relieve oxidative stress in *O. mykiss*.

In fish, complement plays an essential role in activating innate immune responses and clearing potential pathogens [49]. Besides, it can bind to specific sites on the surface of phagocytes to promote phagocytosis [50]. Lysozyme, as an important antibacterial molecule, can lyse the polysaccharide wall of bacteria, thereby preventing pathogen infections and diseases [51]. Lysozyme and complement systems can act synergistically in pathogen clearance [52]. Therefore, lysozyme and complement are widely used as biomarkers for evaluating the innate immune status in fish [9, 53, 54]. In this study, *O. mykiss* fed a diet containing 251.8 g/kg carbohydrate level showed significantly lower mRNA transcription levels of *Lyz* and *C3* than those fed diets containing 50.6-200.9 g/kg carbohydrate levels. Similarly, Zhou et al. [9] indicated that *T. ovatus* fed a diet containing 280 g/kg carbohydrate level had the lowest plasma LYZ activity and C4 content than those fed diets containing 0-224 g/kg carbohydrate levels. Yilmaz [13] demonstrated that 270 g/kg potato starch supplementation negatively affected lysozyme activity in *O. mykiss*. Similar results have reported in *S. fontinalis* and *E. ilishaeformis* fed with a high carbohydrate diet [44, 46]. The findings of this study indicated that high-carbohydrate diet impaired the immune ability of *O. mykiss*.

## 5. Conclusions

It is concluded that 251.8 g/kg carbohydrate level suppressed growth performance, antioxidant capacity, and innate immunity of *O. mykiss*. Besides, 251.8 g/kg carbohydrate level led to liver injury and the occurrence of inflammatory response. A diet containing more than 200.9 g/kg carbohydrate is not efficiently utilized by *O. mykiss* under cage culture with flowing freshwater. Based on the analysis of the quadratic regression equation for WGR, the appropriate dietary carbohydrate requirement of *O. mykiss* was estimated to be 126.2 g/kg.

## Figures and Tables

**Table 1:** Composition and nutrient levels of the experimental diets (g/kg dry matter).

Ingredients	T1	T2	T3	T4	T5
Fish meal	450	450	450	450	450
Soybean meal	20	20	20	20	20
Soy protein isolate	118.1	118.1	118.1	118.1	118.1
Corn starch	50	100	150	200	250
Bone meal^a^	200	150	100	50	0
Fish oil	100	100	100	100	100
Soybean lecithin	20	20	20	20	20
Ca(H_2_PO_4_)_2_	10	10	10	10	10
Vitamin premix^b^	10	10	10	10	10
Mineral premix^c^	10	10	10	10	10
Choline	5	5	5	5	5
Vitamin C	5	5	5	5	5
DL-met	1.9	1.9	1.9	1.9	1.9
Total	1,000	1,000	1,000	1,000	1,000
Nutrient levels^d^					
Crude lipid	154.1	155.7	152.9	157.3	156.8
Crude protein	423.9	426.2	424.0	427.4	425.1
Moisture	94.2	92.9	93.6	97.1	95.5
Carbohydrate	50.6	102.1	151.3	200.9	251.8

^a^Defatted and denitrified bone meal, supplied by Junyou Feed Corporation, Guangzhou, China.^b^ Multi-vitamin (kg^−1^ diet): Vitamin B1 30mg, Vitamin B2 60mg, Vitamin B6 20mg, Nicotinic acid 200 mg, Calcium pantothenate 100 mg, Inositol 100 mg, Biotin 2.5 mg, Folic acid 10 mg, Vitamin B12 0.1 mg, Vitamin K3 40mg, Vitamin A 3 mg, Vitamin D3 0.05 mg, Vitamin E 160 mg.^c^ Multi-mineral (kg^−1^ diet): MgSO_4_∙7H_2_O 1090 mg, KH_2_PO_4_ 932 mg, NaH_2_PO_4_∙2H_2_O 432 mg, FeC_6_H_5_O_7_∙5H_2_O 181 mg, ZnCl_2_ 80 mg, CuSO_4_∙5H_2_O 63 mg, AlCl_3_∙6H_2_O 51 mg, MnSO_4_∙H_2_O 31 mg, KI 28 mg, CoCl_2_∙6H_2_O 6 mg, Na_2_SeO_3_∙H_2_O 0.8 mg.^d^ Measured values.

**Table 2:** Primer information of Real-time fluorescent quantitative PCR.

Gene	Primer sequence(5' to 3')	Genbank no.
*SOD*-F	TGAAGGCTGTTTGCGTGCTGAC	NM_001160614.1
*SOD*-R	CCGTTGGTGTTGTCTCCGAAGG	
*GPX*-F	TCATCATGTGGAGCCCTGTCTG	AF281338.1
*GPX*-R	TCTGCCTCAATGTCACTGGTCA	
*IL-2*-F	GAAACCCAATTCCCAGACTC	AM422779.1
*IL-2*-R	TCCGTTGTGCTGTTCTCCT	
*IL-1β*-F	ACGGTTCGCTTCCTCTTCTACA	AJ245925.2
*IL-1β*-R	GCTCCAGTGAGGTGCTGATGAA	
*IL-8*-F	GTCAGCCAGCCTTGTCGTTGT	NM_001124362.1
*IL-8*-R	CGTCTGCTTTCCGTCTCAATGC	
*Nrf2*-F	GCAGAGGTCTGCCCACCTGAAT	HQ916348.1
*Nrf2*-R	GCCACAAGGCAGGGTGACACTT	
*keap1*-F	GCTACGTGATGTCTGCCCCT	NC_048581.1
*keap1*-R	GGTACCTCATAGCGGCCAGT	
*HO-1*-F	CGCCTACACCCGTTACCTAG	AF099079.2
*HO-1*-R	CTCTCCGCTGCTTAACCCAA	
*TNF-α*-F	GGCGAGCATACCACTCCTCTGA	NM_001124362.1
*TNF-α*-R	AGCTGGAACACTGCACCAAGGT	
*HSP70*-F	GGACGCAGCCAAGAACCAAGT	NC_048591.1
*HSP70*-R	GGCCGTGTCGAGTCGTTGAT	
*C3*-F	GGCCAGTCCCTGGTGGTTA	XM_036955530.1
*C3*-R	GGTGGACTGTGTGGATCCGTA	
*Lyz-*F	GAAACAGCCTGCCCAACT	AF452171.1
*Lyz-*R	GTCCAACACCACACGCTT	
*GR*-F	CTAAGCGCAGCGTCATAGTG	HF969248.1
*GR*-R	ACACCCCTGTCTGACGACAT	
*β-Actin*-F	TACAACGAGCTGAGGGTGGC	AJ438158.1
*β-Actin*-R	GGCAGGGGTGTTGAAGGTCT	

**Table 3:** Effects of dietary carbohydrate levels on growth performance of *Oncorhynchus mykiss.*

	Dietary carbohydrate levels							
	T1	T2	T3	T4	T5	ANOVA	Linear	Quadratic
IBW (g)	25.83 ± 0.42	25.67 ± 0.19	25.50 ± 0.25	25.83 ± 0.25	25.78 ± 0.11	0.88	0.94	0.75
FBW (g)	59.10 ± 1.39^a^	60.98 ± 0.89^a^	59.41 ± 0.78^a^	61.25 ± 1.60^a^	53.94 ± 0.77^b^	0.01	0.08	0.01
WGR (%)	129.03 ± 8.29^a^	137.59 ± 2.54^a^	132.97 ± 1.09^a^	134.11 ± 6.93^a^	109.25 ± 3.24^b^	0.02	0.06	0.01
SGR (%/d)	1.97 ± 0.09^a^	2.06 ± 0.03^a^	2.01 ± 0.01^a^	2.02 ± 0.07^a^	1.76 ± 0.04^b^	0.02	0.06	0.00
SR (%)	97.78 ± 1.11	95.56 ± 2.22	97.78 ± 1.11	94.44 ± 1.11	97.78 ± 1.11	0.35	0.82	0.61
FCR	1.27 ± 0.05^a^	1.25 ± 0.02^a^	1.30 ± 0.02^a^	1.37 ± 0.05^a^	1.59 ± 0.02^b^	0.01	0.00	0.00
FI (%/day)	2.38 ± 0.03^a^	2.35 ± 0.02^a^	2.42 ± 0.02^a^	2.41 ± 0.02^a^	2.24 ± 0.02^b^	0.00	0.08	0.01

Values are presented as mean ± SE, n =3. The superscript small letters in the same row means the significant difference at *P* <0.05.

**Table 4:** Effects of dietary carbohydrate levels on whole body composition (g/kg wet weight) of *Oncorhynchus mykiss.*

	Crude protein	Crude lipid	Moisture
T1	176.3 ± 1.8	122.9 ± 0.6	665.6 ± 3.7
T2	178.6 ± 1.2	121.6 ± 1.0	668.0 ± 4.1
T3	177.2 ± 0.9	122.3 ± 0.9	664.1 ± 5.2
T4	178.1 ± 1.9	121.8 ± 0.8	666.0 ± 2.8
T5	178.3 ± 2.1	122.6 ± 1.2	665.2 ± 3.2
ANOVA	0.46	0.44	0.80
Linear	0.26	0.82	0.68
Quadratic	0.47	0.34	0.92

Values are presented as mean ± SE, n =9.

## Data Availability

All the data in the article are available from the corresponding author upon reasonable request.
